# The breast cancer risk assessment pathway in England: a systems analysis of current challenges and ways to improve

**DOI:** 10.1038/s41416-025-03329-2

**Published:** 2026-01-06

**Authors:** Maria Valasaki, Lily C. Taylor, Victoria G. Woof, Sacha J. Howell, Marc Tischkowitz, Antonis C. Antoniou, Stephanie Archer, David P. French, Juliet. A. Usher-Smith

**Affiliations:** 1 Department of Public Health and Primary Care, University of Cambridge, Cambridge, UK; 2 Division of Psychology and Mental Health, University of Manchester, Manchester, UK; 3 Division of Cancer Studies, University of Manchester, Manchester, UK; 4 Department of Medical Genetics, University of Cambridge, Cambridge, UK; 5 Department of Psychology, University of Cambridge, Cambridge, UK

**Keywords:** Breast cancer, Cancer prevention

## Abstract

**Background:**

In England, NICE guidance recommends enhanced screening and prevention for women at increased breast cancer risk. This study aimed to map current risk assessment pathways for women under 50, explore existing challenges and opportunities for improvement, and assess the healthcare system’s capacity and readiness for potential systemic change.

**Methods:**

Informed by the systems engineering framework and the Consolidated Framework for Implementation Research, we conducted semi-structured interviews and a post-interview questionnaire. Participants were 29 healthcare professionals and policy stakeholders across England with relevant expertise spanning all stages of the breast cancer risk assessment and management pathway. They were purposefully recruited through the research team’s networks and by using the snowball method. Data were analysed by using thematic analysis. Questionnaire data were analysed descriptively.

**Results:**

Participants discussed the challenges of the current pathway and consistently described it as fragmented, with access often influenced more by ethnicity, geographic and socioeconomic factors than by clinical need. Participants identified six key areas where reform is needed, including a standardised national service, developing digital and flexible tools, a shift towards being proactive, division of responsibilities, a need for funding and strengthening skills in risk assessment.

**Conclusions:**

This study demonstrates challenges, variation and missed opportunities in care within the current pathway for breast cancer risk assessment and management in women under 50. The readiness of the system to change is clear, and recommendations are made to support systemic reform, which could contribute to the development of a more standardised, proactive and equitable breast cancer risk assessment and management pathway.

## Introduction

Breast cancer (BC) is the most common cancer worldwide and in women accounts for ~25% of cancers and 15% of cancer deaths [1]. Between 15 and 20% of BC cases occur in women younger than 50 [2]. The global BC incidence rate per 100,000 in this age group has risen by 40% in the past 30 years [3]. Approximately half of cases of BC are estimated to occur in the 12% of the population at greatest risk [4]. In England, the National Institute for Health and Care Excellence (NICE) recommends that women at moderate or high risk of BC should be offered enhanced annual breast imaging starting from age 30 to 40, alongside the offer of preventive medication [5]. Data from prospective cohort studies suggest that annual mammography may reduce mortality from BC in this group by 12–29% [6, 7] and there is high-quality evidence that preventive medication (e.g. tamoxifen) reduces the relative risk of primary BC in younger women (premenopausal or aged <50) by at least a third [8]. Despite these recommendations, a survey of regional genetics units conducted 15 years after the introduction of the NICE guidance found that many women were unable to access enhanced screening [9] and a study of healthcare professionals reported significant barriers to implementing preventive medication [10].

The current pathway for identifying unaffected women at moderate or high risk of BC, eligible for additional screening under age 50, remains heavily dependent on women presenting to primary care with concerns about their family history. Consequently, it is estimated that fewer than 10% of such women are aware of their increased risk [11]. Only 17% of eligible women are offered preventive medication, and fewer than 10% of those actually receive a prescription [12]. Additionally, reliance on self-presentation for risk assessment disadvantages those from deprived backgrounds [13] and likely introduces inequalities.

For the past few years, research has assessed the feasibility and acceptability of proactive risk assessment as a means of changing the current landscape of BC prevention [14–16]. Studies among clinicians and women have shown considerable enthusiasm for this approach [15, 17], while others have evaluated its psychological impact on women, finding it to be relatively low [18, 19]. In line with this emerging direction, the aims of this study were:

- to map out the current BC risk assessment and screening pathways for women under age 50 in England,
- assess capacity and readiness for systemic change and
- identify potential strategies for implementing risk assessment that are most likely to optimise the adoption of BC risk assessment and subsequent uptake of risk-reducing options.

## Methods

### Design

We conducted qualitative interviews, followed by an online post-interview questionnaire, informed by the systems engineering framework [20, 21] and the Consolidated Framework for Implementation Research (CFIR) [22]. The systems engineering framework was chosen for its holistic evaluation across four key perspectives: people, systems, design and risk [13]. We have successfully applied it to the bowel cancer screening pathway [23] and broader literature supports its use in improving healthcare pathways [20]. The CFIR was chosen as it is considered one of the most efficient approaches to gain evidence on barriers and facilitators of potential interventions in different contexts, particularly in healthcare, and considers factors at system, individual and local levels [24]. By combining these approaches, we aimed to develop a holistic understanding of the current pathway and factors influencing BC risk assessment, management and screening, while also identifying actionable insights to optimise the pathway and inform practical and evidence-based improvements to policy and practice.

### Participants and recruitment

Eligible participants were healthcare professionals and policy stakeholders involved in BC risk assessment, management and screening in England. Initially, potential participants from each group as described in Table 1, were purposefully approached [25] through the research team’s professional networks, including—but not limited to—connections with clinical services, Cancer Alliances, the UK Cancer Genetics Group, regional Directors of Breast Screening, the NHS Genomic Medicine Service, etc. Participants were then asked to suggest additional individuals to facilitate snowball sampling [26]. Eligibility, defined here as being actively involved in the field in any area of England (excluding Northern Ireland, Wales and Scotland due to variations in practice), was confirmed by personal communication prior to scheduling the interviews.

**Table 1:** Participants and field of expertise.

Participants’ fields of expertise	Number of interviews	Questionnaire respondents
Primary care		
General practitioners	4	1
Secondary care		
Breast surgeons	4	6
Breast physicians	1	
Oncologists	2	
National breast screening programme		
Radiologists	6	6
Clinical Genetics Services		
Genetic counsellors	5	4
Family history clinics		
Nurses	5	5
Commissioning		
Commissioners	2	2
**TOTAL**	**29**	**24**

The bold values suggest the total number of participants in the interviews and the questionnaire.

Participation was voluntary, and all participants were approached through emails that included an information sheet outlining the study’s aims and their involvement. Informed consent was obtained either electronically or verbally prior to the start of the interview. We aimed to recruit a diverse sample of 15–25 participants that was based on our assessment of the needs of the study, i.e. the range of expertise of healthcare professionals involved, the information participants could provide based on their expertise, and the complexity of the current pathway. Recruitment was stopped when sufficient information power had been reached [27].

### Data collection and materials

Data were collected between November 2024 and January 2025, through online semi-structured interviews that lasted approximately an hour each. Most of them were one-to-one, though in three cases, participants invited colleagues to attend, with valuable insights. While designing the interview guides, we developed a map of the current pathway along with potential ways in which it could be optimised, based on clinical and topic expertise within the research team. The current pathway map was split into two parts: (a) access to risk assessment and referrals from primary to specialist care; and (b) risk management. The interview guide (Appendix 1) reflected this structure with three sections: (a) access to risk assessment and ways to improve it in primary care, including offering it during NHS Health Checks, chronic condition reviews, cervical screening, via GP letters, or through community, religious groups, or pharmacies; (b) risk categorisation and management; and (c) potential pathway changes, such as primary or secondary care leading the risk assessment. Interview questions were informed by the CFIR, offering a structured way to explore organisational context, external influences, stakeholder roles and implementation processes.

Following the analysis of the qualitative data, the main findings were summarised and used to develop a brief online questionnaire, which was distributed to the same participants and completed anonymously. The purpose of this questionnaire was not to generate quantitative data, but to validate and strengthen the initial interview findings, avoid interpretative bias [28] and to identify the recommendations with the greatest overall support, as presented in the discussion of this article. The questionnaire was divided into three parts: (a) challenges of the current pathway; (b) suggested changes to the current pathway; and (c) a revised map of the current pathway based on participant interview insights. Throughout the questionnaire, participants were asked to select their answer from a 5 Point Rating Scale (strongly agree to strongly disagree).

### Analysis

Interviews were recorded and transcribed verbatim by an external transcription company, transcripts were pseudonymised and were analysed using NVivo V.12. We adopted a deductive thematic analysis approach [29, 30] as a ‘top-down’ way of identifying codes, categories and themes within the dataset based on existing literature and theory. The coding process was guided by the predefined structure of the interview guide, but iterative refinement allowed for the identification of nuances within participants’ views. An initial codebook was developed by two members of the team (MV and LT), both non-clinical researchers with extensive experience in qualitative research. The codebook was used to support the development of themes in conjunction with a third, clinical researcher with experience in qualitative research (JUS).

Data were analysed from all participants collectively and from each group separately and compared between groups. As data analysis progressed, themes were adjusted to better reflect the complexities of the pathway and the ways to improve it, which are collated in this article as a list of recommendations. Results from the questionnaire were analysed descriptively and reported as percentages for each response option in Tables 2 and 3 in the results below. In these analyses, ‘Strongly agree’ and ‘Agree’ were combined to give the percentage of participants who agreed with individual statements or recommendations.

**Table 2:** The current pathway as a fragmented pathway with illustrative quotations and questionnaire data.

Sub-themes	Illustrative quotations	% of participants agreeing with these statements in the survey
A postcode lottery	‘…we have had problems where one sister lives in the X area, another sister might live in Y or Z or somewhere and they’ve not been able to access the screening that their sister has. So, for example, we might have started their mammograms at thirty-four the sister can’t access any screening locally until forty. So, we know that there are national variation in the way that the guidelines are interpreted. So, it’s very much a postcode lottery, I would say overall.’, Family history clinic 3 ‘…because at the moment there is no specific national funding for that, and so for most services are, you know, offering that based on some local regional arrangement and I think it would be preferrable to prevent a postcode lottery, that that was nationally funded. Because at the moment we know that there’s considerable regional variability and how moderate and high-risk breast screening is provided’, Genetics counsellor 3 ‘Interviewer: So, who discuss risk reducing medication? Participant: It’s not overly discussed at this Trust; we work under the umbrella of X and it’s not overly something that is normally given as advice from them for us to do’, Family history clinic 5	The current pathway is a postcode lottery, as different services and varying age criteria (for women under 50) for specific services (e.g. mammograms) exist across different regions 63.7%
Patchy	‘I think it’s patchy. As a woman with a family history, I think it very much depends who you meet on your travels whether or not you’ll get picked up and get the appropriate management. That’s the honest opinion’, Secondary Care Breast Surgeon 2 ‘I suspect it’s about as patchy as everything else in this world. So, I mean there is two acts of access, there is access to a service in that there might not be a service [….] patchy provision a service and I suspect that there is even more socioeconomic and race access to these services because of all the usual socioeconomic and racial ethnic issues around access’, Screening 4 ‘At the beginning I don’t think GPs even want to prescribe… That’s a whole can of worms, I mean… So we tell our patients about risk-reducing medication and then we bat it back for the GPs to prescribe. Some of the GPs don’t want to prescribe it, they don’t really know an awful lot about it, and therefore they feel actually we in secondary care should be prescribing it…’, Family history clinic 1	Patchy can be used as a term to describe systemic inconsistencies and variations in the pathway (i.e. differences in individual GP knowledge on risk assessment and differences in access amongst socioeconomic groups) 86.3%
Opportunistic	‘But at the moment, that person has to come and say, ‘I’m concerned’, and then the GP has to refer them, so that means that it is opportunistic and it’s not systematic, so it means that it’s going to be a bias towards those people who are higher education, more well off, and it’s going to be less equitable for those people who are poorer and don’t know about these things and don’t know about access’, Oncologist 2 ‘So first of all it’s obviously reliant on self-presentation. So we absolutely know from data to our service that we have a bias select of cohort of predominantly white British patients. If you don’t speak English as a first language, even if you’re white, if you come from Poland or Spain or anything like that, we significantly… ’, Genetics counsellor 2 ‘Oh yeah, yeah. [Reading slide]. Yes, so I’d say that we don’t typically discuss tamoxifen or chemo prevention with all moderate risk, because I think NICE guidance is too considered in that group, and I don’t think, so I don’t think it’s discussed with everyone.’, Genetics counsellor 3	The current pathway is opportunistic because it depends on women’s self-presentation 90.8%

**Table 3:** The need for systemic reform with illustrative quotations and questionnaire data.

Sub-themes	Illustrative quotations	% of participants agreeing with these statements in the survey
Standardised national service	‘I think there needs to be a national strategy for family history and for breast screening generally and I think everybody needs to ideally be doing the same thing because it’s okay, we say, oh well in X [Hospital] we do this, X [Hospital] we do that and we say this a lot because we are doing things perhaps differently than some other places. But I can give so many examples of variation in practice’, Family history clinic 3 ‘Ideally, I think that moderate and high-risk screening should be incorporated into the national breast screening programme, and so I think that they should just be part of the national breast screening programme, but that isn’t the case at the moment’, Genetics counsellor 3 ‘It looks after population risk and very high risk, that comes under your national screening programme, overseen nationally by NHS England but the risk in between, the moderate and the high risk is not overseen by NHS England, that is a locally managed service which makes no sense.’, Screening 6	There should be a national strategy for risk assessment and family history, ensuring a standardised approach for all 91%
Developing digital and flexible tools	‘I mean, if there was a hub with all the region feeding into a massive… […] where they were screening for you know, other cancers and breast, and … sent their patients into a hub and then… they would have the most up to date assessment tools and organise the screening. I guess that would… Maybe it’s a money-saving idea’, Family history clinic 1 ‘…the GPs don’t need to be doing because it could be done at a pharmacy, it could be done … for people filling in their tax return, they don’t need to have an accountant to do it anymore, they can just do it online and we need to make it more accessible and easier for people to do that’, Screening 5 ‘So I agree that, I think it’s probably a mixed methods model. So, I think, certainly groups of people who definitely would prefer electronic methods of being contacted, rather than letter, but I think for other groups as you mentioned, people whose maybe first language isn’t English, you know, maybe then some of the alternative ways you suggested’, Genetics counsellor 3	There is an emerging need for digitalisation of the pathway, i.e. digital monitoring, tools for quick triaging, digital family history questionnaires 100%
A shift towards being proactive	‘I think to improve the situation with the current pathway we could promote awareness about family history addressing some common misconceptions […] So, all women get posted a questionnaire for their 35th birthday and proactively asked about family history and whether they’ve got any concerns. At the moment the whole pathway is very opportunistic and the people who are more likely to voice their concerns are the ones who are more likely to present to services which isn’t hugely fair of the underserved section of society’, Genetics counsellor 5 ‘That’s exactly what you need. Yes, and also as you say going through the religious groups, community groups, it’s about breaking down barriers…’, Oncologist 2	There is an emerging need for shifting towards a proactive system that would be well-structured and actively reach and support women of all groups 91%
Division of responsibilities	‘You know, you need to understand that data to be managing these patients. That sits somewhere between the secondary care and genetics. It should definitely not sit in primary care. I speak as someone who uses this all the time… There’s risk of harm in identifying the wrong people…’, Secondary Care Breast Surgeon 2 ‘Moderate risk patients could then be managed in secondary care by family history clinics and they would be able to have discussions about risk reducing medication, and also a range, appropriate screening. And then again I think that the higher risk patients should be managed through a combination of family history and specialist genetic clinics’, Genetics counsellor 3 ‘At the beginning I don’t think GPs even want to prescribe… That’s a whole can of worms… So we tell our patients about risk-reducing medication and then we bat it back for the GPs to prescribe. Some of the GPs don’t want to prescribe it, they don’t really know an awful lot about it, and therefore they feel actually we in secondary care should be prescribing it.’, Family history clinic 1	Family history and CGS are best placed for doing risk assessment 82%
Need for funding	‘I think we’re at the best point in time to make those changes. The logical side or the pragmatic side of me is saying there is just not enough funding but I think mentally everybody is ready for that change, more than ever’, Family history clinic 2 ‘There is a similar pressure in breast oncology clinics, but I do think that there needs to be funding and provision and part of the problem we have at the moment is there is no funding and provision and that’s why it’s falling in Noman’s land and that’s why it’s weird across the country as to who takes responsibility for this service. Another example in X, the oncologists do the family history risk assessment. When you go to Z they have their own service run by nurses that they’re cancer nurses who belong to the breast surgical department. So it’s very, very, variable…’, Screening 2	Any improvements to the pathway will require additional funding, particularly in family history and CGS. 98%
Strengthening skills in risk assessment	‘I think the other place is GP education. So if we could go into GP practices or we do education and training events… We need to advertise that the service exists. We could let individual GP practices have some autonomy about how they could make their patient population more aware of genetic cancer risk and the enhanced surveillance programmes and that they were entitled to this service.’, Genetics counsellor 2 ‘I think identification in primary care or public understanding of what constitutes a risky family history scenario. More awareness in breast units because I think unless you’ve got a particular interest in it, not all of my colleagues would know who require extra screening and who doesn’t.’, Breast Surgeon 4	Clinicians in primary care and secondary care will need education about family history and risk assessment 96%

### Ethical approval

Ethical approval was obtained prior to the study by the Social Sciences Research Ethics Committee [HSSREC] of the University of Cambridge. HSS REC reference number: 24.376.

## Results

We contacted 44 individuals, of whom 29 participated in the research: 20 were recruited through the research team’s networks, and 9 through the snowball method. 24 out of 29 participants completed the questionnaire. Participants were based in the East of England (*n* = 12), Southwest (*n* = 2), Southeast (*n* = 9) and Northwest (*n* = 6) regions. In addition to covering all stages within the risk assessment and screening pathway (Table 1), individuals had dual roles, including members of the National Screening Committee, the NICE Guideline Development Group for the current guidelines on Familial BC, etc. Aside from varying knowledge of pathway details, participants reported similar challenges and needs for improvement. Findings are presented collectively, with individual expertise noted alongside quotations. The results are presented within two major themes: [1] The current pathway as a fragmented pathway, and [2] the need for systemic reform. Equity emerged as a cross-cutting theme, with related considerations reported within each main theme.

### The current pathway as a fragmented pathway

**Fig. 1:**
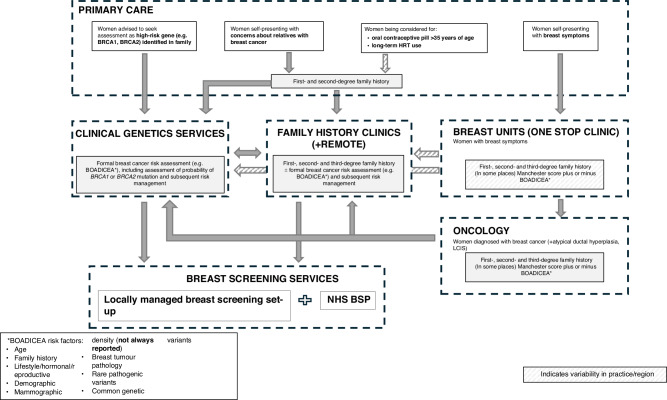
Final map of the current pathway for risk assessment and management.

Table 1 shows the final map of the current pathway for risk assessment and management following the interviews and additional questionnaire comments. Overall, participants described a pathway aligned with NICE guidelines [2], with clinicians in primary care taking a first- and second-degree family history from women presenting with concerns about their family history and referring women with known high-risk genes in the family to Clinical Genetics Services (CGS) and those meeting the family history criteria to CGS or family history clinics. Assessment for women with breast symptoms or who are being considered for the oral contraceptive pill or long-term HRT was more variable. Oncology clinics usually assess risk by taking a detailed family history and then referring to either family history or CGS. Risk assessment in breast units was less consistent. Women have their risk assessed in family history or CGS using one of several risk models, with specific management of those at moderate or high-risk variables and coordinated locally (Fig. 1).

While some participants described pockets of good practice, the current pathway was described as fragmented. Fragmentation stems from differences in regional policies, resource allocation and service delivery, leading to inequalities in healthcare services. This theme entails three sub-themes which contribute to a more comprehensive understanding of the fragmentation of the current pathway: being a postcode lottery; patchy; and opportunistic. These are described below, and illustrative quotations and questionnaire data are provided in Table 2.

### A postcode lottery

The concept of a ‘postcode lottery’ arose from a common view amongst participants that accessing risk assessment and the subsequent management options are dependent on the region of the country. They described not only differences in the age range for eligibility for mammograms in different regions, but also in some regions a total lack of services. Indeed, one participant gave an example where a member of one family can access mammograms, whereas another member of the same family with the same estimated risk category cannot because they live in another part of the country. A similar situation was seen with preventive medications, with discussions around the risks and benefits not taking place in all regions. This ‘postcode lottery’ was thought to be secondary to a lack of national funding, leading to reliance on regional decision-making and priorities, which creates inconsistencies in service availability.

### Patchy

While the ‘postcode lottery’ perspective reflects disparities primarily due to geographical funding decisions, the term patchy was used to describe a broader, more systemic critique, incorporating issues of provider awareness, standardisation and social inequalities. As well as this variation between regions in what services are offered, participants recognised the challenge and variation in experience that women face accessing and navigating the current pathway, with several explicitly using the term ‘patchy’.

This characteristic was also given as there is inconsistency and discontinuity in the quality of services. Participants emphasised that care depends on ‘*who you meet on your travels*’ (Breast Surgeon), and that there is a lack of standardised procedures. Such issues within the system were further reinforced by one participant describing the representation of the pathway as ‘*In practice - it is not joined up as suggested by the figure*’ (anonymised in the questionnaire).

Variation was particularly seen in primary care, where the limited time for consultations and lack of awareness amongst some GPs can impact whether women are offered appropriate risk assessment and referral. Preventive medication also emerged as a particularly patchy aspect of the pathway. Not only are there no specific guidelines about initiating prescriptions and following up with women, but there were also examples given where there had been pushback from GPs due to a lack of expertise.

### Opportunistic approach

Characterising the current pathway as ‘opportunistic’ arose from the observation that women need to self-present with concerns about breast symptoms or BC risk to be assessed; only those who are proactive or informed about risk assessment will benefit. During the interviews, it was noted repeatedly that this creates a bias towards wealthier, higher-educated, English-speaking individuals who are more likely to advocate for themselves. Language barriers, socioeconomic status and ethnicity all emerged as significant determinants of access to this service, with low attendance among ethnic minorities thought to be particularly linked to a system that relies too heavily on individual knowledge, rather than proactive outreach efforts.

Overall, the current pathway for risk assessment and management for women at above population risk was felt to depend more on social and systemic factors than on clinical need.

### The need for systemic reform

As participants described the challenges of the current pathway, they discussed the need for systemic reform, which emerges from the necessity of making fundamental, structural changes as a way of creating a more efficient, equitable and coordinated system, and as a way of tackling fragmented care, unequal access, inconsistencies and outdated processes. This theme entails six sub-themes, which represent the key areas where reform is needed: standardised national service, developing digital and flexible tools, a shift towards being proactive, division of responsibilities, need for funding and strengthening skills in risk assessment. Table 3 provides illustrative quotations and questionnaire data for each of these six sub-themes.

### The need for a standardised national service

The lack of a standardised national service was felt to contribute to many of the challenges within the current pathway. During the interviews, participants consistently spoke about the need for a national service that would ensure a standardised approach for all, prevent the ‘*many examples of variation in practice*’ (Family history nurse), and provide continuity of care for women, regardless of where they live. Additionally, several participants suggested that the additional screening for moderate and high-risk women should be incorporated into the NHS Breast Screening Programme (BSP). Population risk and very high-risk women already come under the NHS BSP. But moderate and high-risk groups fall outside national oversight and are managed locally, which ‘*makes no sense*’ (Radiologist).

### Developing digital and flexible tools

Digitalisation emerged as a consistent requirement and underpinned many other suggestions for the improvement of the system. In the longer term, participants suggested there could be automated systems that, instead of relying on letters and emails to women, could match existing patient data against risk-based protocols and direct women into the appropriate pathways. In the shorter term, a national hub for online risk assessment and a dataset for women at above population-level risk was suggested as a cost-saving and efficiency-driven approach; this could provide standardised, up-to-date assessment tools and ensure equitable access. Digital tools could improve access to risk assessment by enabling family history questionnaires to be completed in alternative settings, reducing reliance on clinicians and making the process more patient-centred.

In the context of patient-centred care, participants talked about women’s potential needs and ways to address them, e.g. having a hotline for women to seek advice at their convenience. They also felt that non-native English speakers may require alternative communication strategies (e.g. interpreters or community outreach), whereas others might prefer digital methods (e.g. emails, text messages) over traditional letters.

### A shift towards being proactive

Describing the system as opportunistic directed the discussion towards the need for a more proactive approach. While there was no consensus on the optimal age to begin risk assessment, there was agreement on the need for a baseline age. Within primary care, of the options presented to participants for proactive risk assessment, NHS health checks from age 40 were considered the best opportunity. This was despite recognition that this would still miss a lot of women due to low and, in places, inequitable uptake. Approaching women during health checks for chronic conditions or cervical screening was less favoured than NHS Health Checks due to the danger of increasing anxiety. It was also commonly accepted that there is a need for the system to include approaches tailored towards women from underserved groups. This could be achieved through religious and community groups; participants voiced concerns that attendance in such settings could also be limited. Similarly, introducing risk assessment in pharmacies was seen as feasible, though it was not a common preference. Outside primary care, participants embraced the idea of introducing risk assessment when women present to secondary care with breast symptoms.

### Division of responsibilities

A key focus was on where the system risk assessment and management would best be conducted. When asked if primary care should have a larger role in risk assessment, all expressed that although it could be a possibility, there is no capacity (time, skills and staff). Conversely, participants felt that family history clinics and CGS would have the capacity and expertise. Moreover, participants felt that there would be scope for an increase in capacity for women in family history clinics and in screening if the pathway was optimised, unlike primary care, where capacity would still be limited.

Similar concerns were raised regarding the prescription of preventive medication, which was described by one participant as a ‘*can of worms*’ (Family history nurse). There was no consensus on who should lead prescribing—some supported primary care, others cited its limited capacity and favoured family history or CGS. While family history nurses noted they cannot prescribe, all participants agreed that trained professionals are essential for risk assessment, counselling and prescribing.

Participants described the need to divide responsibilities between primary care, family history clinics, and CGS to support a structured, risk-stratified approach and ensure expert care at all risk levels. Participants suggested that primary care continue its triage role and manage women at population risk. The most preferred suggestion was that moderate-risk women be managed in family history clinics, while high-risk management was widely viewed as a shared responsibility between family history clinics and CGS. Adapting to existing resources and structures, rather than reinventing them, was seen as key. A national remote service with trained staff for risk assessment was considered an ideal solution.

### Need for funding

Participants consistently spoke about the need for an increase in funding. Limited funding was felt to be the cause of many of the inconsistencies in service provision across different locations and the fragmentation of the pathway. An increase in funding was felt to be needed if optimisation was the ultimate goal, but participants recognised that finding additional funding within the NHS is difficult and felt that re-organisation of the system by using the same funding more efficiently could be possible. Funding should be prioritised to enhance capacity in family history clinics, screening, CGS and secondary care.

### Strengthening skills in risk assessment

Participants highlighted the pressing need for increased education and awareness around risk assessment, particularly within primary care. The importance of integrating education into GP practices was emphasised, suggesting that local autonomy could empower practices to better engage women and promote tailored outreach to improve patient access to risk assessment and screening. Similarly, education and increased awareness are also needed in secondary care for staff not familiar with risk assessment, because, as was mentioned by one participant, unless someone has a special interest in risk assessment and family history, they are not aware of it.

## Discussion

Through engagement with 29 experts representing all stages of the BC risk assessment and management pathway across England, this study highlights significant challenges in the current pathway and the need for improvement. Participants were supportive of BC risk assessment and there were positive aspects -such as good practices in certain regions and participants’ enthusiasm for risk assessment and its importance in cancer prevention. However, the current pathway was consistently described as a postcode lottery, patchy and opportunistic; access to risk assessment and management was often influenced more by ethnicity, geographic and socioeconomic factors than by clinical need.

The enthusiasm for BC risk assessment seen in this study has been reported previously among both general population women [15] and healthcare professionals [17]. However, although not reported in this context previously, the description of the system as patchy and as a postcode lottery by the participants is not unique to BC risk assessment and management. The term ‘postcode lottery’ has been used since the mid-1990s to refer to differences in healthcare between different geographic areas that appear arbitrary and unlinked to health need [31]. Evidence of such differences has been reported across multiple conditions, e.g. diabetes [32], and with clinical outcomes, e.g. in the case of out-of-hospital cardiac arrest [33]. Within BC specifically, the existence of variation between regions is supported by a survey of regional genetics units 15 years after the introduction of the guidance, which found that many women were unable to access enhanced screening [9]. Our findings suggest that this is due to screening being managed locally, leading to a lack of oversight regarding who gets screened, at what age and how often. We additionally identified variation in the care women receive depending on which healthcare professional(s) they meet, especially in primary care, where knowledge around BC risk assessment was felt to be particularly variable. Concerns about the opportunistic pathway, where self-presentation favours informed women and disadvantages ethnic minorities, low-income groups, or those facing language barriers, have also been previously reported, both in the context of BC risk assessment [13] and in other healthcare settings [34].

Based on these descriptions of the challenges in the current system and suggestions from participants on ways it could be improved, we developed ten recommendations (Table 4). The first three relate to the introduction of national systems and processes and include recommendations for a standardised national risk assessment pathway, incorporation of enhanced screening for women at above-population-level risk into the national BSP, and a national register to enable systematic recall and recording of outcomes. Similar changes have recently been made for individuals with Lynch syndrome [35–37] and could act as templates for BC.

**Table 4:** Recommendations.

Recommendations	
Standardised national service	
1	There should be a national strategy for risk assessment and management, ensuring a standardised approach for all.
2	Additional screening for women at moderate and high risk should be incorporated within the NHS Breast Screening Programme.
Digitisation and flexible tools	
3	There is a need for a national register with centrally stored data for women at above-population-level risk.
4	There should be flexible and accessible digital tools alongside the offer of support or paper versions to improve access to risk assessment.
A shift towards being proactive	
5	There is an emerging need for shifting towards a proactive system to improve equity of access to risk assessment.
6	Consideration should be given to offering risk assessment within NHS Health Checks, at cervical screening, via religious or community groups and when women present to secondary care with breast symptoms.
Division of responsibilities	
7	Moderate-risk women should be managed by specialist secondary care clinics and high-risk women should be managed between those clinics and CGS.
8	There is a need for clear shared-care pathways for counselling and initiation of preventive medication.
Funding	
9	Funding should be prioritised to enhance capacity in family history clinics, screening, CGS and secondary care.
Strengthening skills in risk assessment	
10	Ensure clinicians in primary care and those in secondary care, seeing women with breast symptoms, have access to training around risk assessment and management.

The fourth recommendation suggests there should be flexible and accessible digital tools alongside the offer of support or paper versions to improve access to risk assessment. Digitalisation within the NHS has been a priority in recent years [38], and research has shown that failing to do so contributes to further fragmentation of the system [39]. In this study, all participants emphasised the importance of digital tools to improve access to risk assessment for women, regardless of location. The value and importance of digital tools have been highlighted in multiple healthcare-related fields [34, 39, 40], with online risk assessment tools recommended and previous studies in BC risk assessment demonstrating the need for such tools [41]. However, tools need to be inclusive, user-friendly, patient-centred and adjusted to women’s needs [42, 43] to avoid contributing to inequalities [44]. In this research, participants suggested providing telephone helplines and offering paper and online versions of family history questionnaires.

Participants in this study also suggested the need to shift towards being proactive to identify women at above-population-level risk. A recent review of the evidence surrounding proactive BC risk assessment within primary care found that risk assessment for women under 50 currently satisfies many of the principles for screening [45]; validated risk models are available that can accurately identify at-risk women, and there are evidence-based management options that reduce BC incidence, morbidity and mortality in these women [2]. Furthermore, data from the NHS BSP suggest that providing BC risk estimates to women aged 47–73 does not increase anxiety and does not produce false reassurance among those at lower risk [19]. However, there remain several uncertainties. These include how best to invite women, especially those who belong to underserved groups, what the uptake of BC risk assessment and management options amongst these women would be, the acceptability of BC risk assessment amongst women and clinicians, and whether proactively offering risk assessment would be cost-effective. Some of these uncertainties will be addressed through ongoing studies, including the BCAN-RAY [15] and CanRisk-GP [14], and studies which are inviting women aged 30–39 and 40–50 years, respectively, for a risk assessment via a letter from their GP.

There was no clear consensus amongst the participants of this study on when or how women should be invited, similar to previous research. However, all agreed that risk assessment should be offered at or before the age of 40 [15, 46] and emphasised the need for approaches to ensure that they consider the needs of different population groups to reduce health inequalities. The most promising routes for offering risk assessment were felt to be within NHS Health Checks or in secondary care when women present with breast symptoms. Incorporating cancer risk discussions into NHS Health Checks within primary care has previously been shown to be feasible and acceptable to patients and clinicians [47–49]. With more NHS Health Checks being delivered outside general practice and in the community, this could also overcome some of the challenges of capacity in general practice and capitalise on key points of contact within community and religious groups to improve equitable uptake. It is likely that a range of approaches will be needed [50].

The next two recommendations address the division of responsibilities in the healthcare system. Participants highlighted the need for a structured, risk-stratified approach to BC risk assessment, with tailored care based on women’s risk level. While primary care was seen as suitable for having a larger role, consistent with current studies [51], all felt that there is currently a lack in capacity (time, skills, staff) to lead risk assessment and management, and instead suggested that focus should be on initial assessments with referrals to specialised services for confirmation of risk and management. Conversely, participants agreed that family history clinics and CGS are well placed for conducting risk assessment and suggested that family history clinics should manage moderate-risk women, and high-risk women should receive care across both family history clinics and CGS. However, with the move toward comprehensive risk assessment, up to 70% of women may not have a family history of BC; therefore, it may be beneficial to further map the current remit of these clinics and consider renaming them to better reflect their broader scope.

Participants additionally recognised the current gap in the pathway regarding preventive medication and related guidelines and responsibilities. Not only is there no standardised approach to preventive medication, but when discussions around it take place in family history clinics or CGS, inconsistencies around whose responsibility it is to initiate prescriptions and provide follow-up care are prevalent[16]. There is a need for clear shared-care pathways for the counselling and initiation of preventive medication.

The final two recommendations relate to the need for additional funding and access to training around risk assessment and management for clinicians in primary care and those in secondary care seeing women with breast symptoms. Funding is needed not only to develop and implement new technologies but also to increase capacity at all steps of the pathway. The need for training, particularly for primary care clinicians, has been identified previously [51, 52]. Initiatives to provide such training are ongoing and include resources across relevant NHS and professional colleges’ websites, e.g. the Genomic Education programme and bespoke training for BC risk assessment [50].

### Strengths and limitations

To our knowledge, this is the first study to comprehensively describe the current BC risk assessment, management and screening pathway in England for women under 50 years of age. We recruited a large and diverse sample of health professionals, representing a range of fields of expertise and geographical regions. Our use of semi-structured interviews, guided by a systems-based approach and the CFIR, allowed participants to provide in-depth insights into the current pathway and identify potential strategies for improvement. The inclusion of a post-interview questionnaire further enabled us to validate the findings from those interviews [53] and develop recommendations for change that align with participants’ views. Consideration of the whole pathway as a system also meant that we were able to map out and consider changes across all elements of the patient journey rather than considering individual components of the healthcare system in isolation, as has been done previously [51, 54]. This was particularly important when considering questions around capacity and patient flow within the system.

However, some limitations should be acknowledged. Firstly, although recruitment continued until sufficient information power was reached, participants were drawn from our professional networks and likely represent those most engaged with BC risk assessment; their views may not reflect others across the pathway. We also did not employ a formal consensus approach to developing the recommendations. Lastly, while this research covers the entire pathway, time constraints limited the extent to which the details of participants’ suggested improvements could be fully explored during the interviews.

### Implications for research and practice

Overall, the findings from this study clearly demonstrate challenges, variation in care and missed opportunities within the current pathway for BC risk assessment and management in women under 50 years of age. These challenges are systemic and interconnected and participants consistently highlighted the need to improve access and standardise care at every step of the pathway. Some of the recommendations to improve the pathway will require system reform and substantial investment, both in terms of time and funding, and so will require engagement with policy makers and commissioners. Others highlight research needs, including when, where and how to best offer risk assessment to reach women equitably, evidence of the impact and cost-effectiveness of proactive risk assessment, and the development of clear pathways for preventive medication. In the meantime, local services should be encouraged to review and optimise their pathways and make best use of existing resources.

## Supplementary information

Interview Guide

## Data Availability

The dataset supporting the conclusions of this article (pseudo-anonymised transcripts) and study materials (protocol, participant information sheet, consent form) are available via the University of Cambridge Data Repository [10.17863/CAM.117505]. Formal requests for access will be considered via a data-sharing agreement that indicates the criteria for data access and conditions for research use and will incorporate privacy and confidentiality standards to ensure data security.

## References

[1] Sung H, Ferlay J, Siegel RL, Laversanne M, Soerjomataram I, Jemal A, et al. Global Cancer Statistics 2020: GLOBOCAN Estimates of Incidence and Mortality Worldwide for 36 Cancers in 185 Countries. CA Cancer J Clin. 2021;71:209–49. 33538338 10.3322/caac.21660

[2] Cancer Research UK. Breast cancer statistics. https://www.cancerresearchuk.org/health-professional/cancer-statistics/statistics-by-cancer-type/breast-cancer. Accessed May 2025.

[3] Zhao J, Xu L, Sun J, Song M, Wang L, Yuan S, et al. Global trends in incidence, death, burden and risk factors of early-onset cancer from 1990 to 2019. BMJ Oncol. 2023;2:e000049. 10.1136/bmjonc-2023-000049 PMC11235000 39886513

[4] Pharoah PDP, Antoniou A, Bobrow M, Zimmern RL, Easton DF, Ponder BAJ. Polygenic susceptibility to breast cancer and implications for prevention. Nat Genet. 2002;31:33–6. 11984562 10.1038/ng853

[5] Familial breast cancer: classification, care and managing breast cancer and related risks in people with a family history of breast cancer clinical guideline. 2013. Available from: www.nice.org.uk/guidance/cg164. 31940157

[6] Grimm LJ, Avery CS, Hendrick E, Baker JA. Benefits and risks of mammography screening in women ages 40 to 49 years. J Prim Care Community Health. 2022;13. https://journals.sagepub.com/doi/10.1177/21501327211058322. 10.1177/21501327211058322 PMC8796062 35068237

[7] Evans DG, Thomas S, Caunt J, Burch A, Brentnall AR, Roberts L, et al. Final results of the prospective FH02 mammographic surveillance study of women aged 35–39 at increased familial risk of breast cancer. EClinicalMedicine. 2019;7:39–46. 31008449 10.1016/j.eclinm.2019.01.005 PMC6472550

[8] Fisher B, Costantino JP, Wickerham DL, Cecchini RS, Cronin WM, Robidoux A, et al. Tamoxifen for the prevention of breast cancer: current status of the National Surgical Adjuvant Breast and Bowel Project P-1 study. J Natl Cancer Inst. 2005;97:1652–62. 16288118 10.1093/jnci/dji372

[9] Evans DG, Edwards M, Duffy SW, Adlard J, Ahmed M, Barwell J, et al. Sporadic implementation of UK familial mammographic surveillance guidelines 15 years after original publication. Br J Cancer. 2020;122:329–32. 31761901 10.1038/s41416-019-0631-2 PMC7000386

[10] Smith SG, Side L, Meisel SF, Horne R, Cuzick J, Wardle J. Clinician-reported barriers to implementing breast cancer chemoprevention in the UK: a qualitative investigation. Public Health Genom. 2016;19:239–49. 10.1159/000447552 27399355

[11] Evans DG, Brentnall AR, Harvie M, Dawe S, Sergeant JC, Stavrinos P, et al. Breast cancer risk in young women in the national breast screening programme: implications for applying NICE guidelines for additional screening and chemoprevention. Cancer Prev Res. 2014;7:993–1001. 10.1158/1940-6207.CAPR-14-0037 25047362

[12] Curtis HJ, Walker AJ, Goldacre B. Impact of NICE guidance on tamoxifen prescribing in England 2011-7: an interrupted time series analysis. Br J Cancer. 2018;118:1268–75. 29681615 10.1038/s41416-018-0065-2 PMC5943266

[13] Usher-Smith JA, Tooth G, Follows A, Badran AR, Youngs A, Forman A, et al. Patterns of referrals to regional clinical genetics services for women potentially at above-population level risk of breast cancer. BJC Rep. 2024;2:2. 10.1038/s44276-023-00027-5 PMC11523992 39516250

[14] Usher-Smith J, Donoso FS, Stylianou L, Lafont A. A feasibility study of offering breast cancer risk assessment to women through general practice. London, UK. http://isrctn.com/.

[15] Hindmarch S, Howell SJ, Usher-Smith JA, Gorman L, Evans DG, French DP. Feasibility and acceptability of offering breast cancer risk assessment to general population women aged 30-39 years: a mixed-methods study protocol. BMJ Open. 2024;14:e078555. 10.1136/bmjopen-2023-078555 PMC10806663 38199637

[16] French DP, Woof VG, Ruane H, Evans DG, Ulph F, Donnelly LS. The feasibility of implementing risk stratification into a national breast cancer screening programme: a focus group study investigating the perspectives of healthcare personnel responsible for delivery. BMC Womens Health. 2022;22:142. 10.1186/s12905-022-01730-0 PMC9063090 35501791

[17] Roux A, Hervouet L, Di Stefano F, French DP, Giordano L, Ritchie D, et al. Acceptability of risk-based breast cancer screening among professionals and healthcare providers from 6 countries contributing to the MyPeBS study. BMC Cancer. 2025;25:483. 10.1186/s12885-025-13848-z PMC11910845 40089664

[18] Lambert-Côté L, Turgeon A, Blackmore KM, Chang A, Antoniou AC, Bell KA, et al. Psychological and emotional impacts of communicating breast cancer risk using multifactorial assessment with polygenic risk score: findings from PERSPECTIVE I&I. Genet Med. 2025;27:101453. 10.1016/j.gim.2025.101453 40365755

[19] French DP, Southworth J, Howell A, Harvie M, Stavrinos P, Watterson D, et al. Psychological impact of providing women with personalised 10-year breast cancer risk estimates. Br J Cancer. 2018;118:1648–57. 29736008 10.1038/s41416-018-0069-y PMC6008295

[20] Komashie A, Ward J, Bashford T, Dickerson T, Kaya GK, Liu Y, et al. Systems approach to health service design, delivery and improvement: a systematic review and meta-analysis. BMJ Open. 2021;11:e037667. 10.1136/bmjopen-2020-037667 PMC7817809 33468455

[21] Clarkson J, Bogle D, Dean J, Tooley M, Trewby J, Vaughan L, et al. Engineering better care: a systems approach to health and care design and continuous improvement. 2017 [cited 2023 Oct 16]. Available from: https://raeng.org.uk/media/wwko2fs4/final-report-engineering-better-care-version-for-website.pdf.

[22] Damschroder LJ, Aron DC, Keith RE, Kirsh SR, Alexander JA, Lowery JC. Fostering implementation of health services research findings into practice: a consolidated framework for advancing implementation science. Implement Sci. 2009;4:50. 10.1186/1748-5908-4-50 PMC2736161 19664226

[23] Moorthie S, Taylor L, Dennison R, Usher-Smith J. A systems based qualitative analysis exploring the potential to implement risk stratified bowel cancer screening in England. BMC Health Serv Res. 2025;25:226. Available from: https://bmchealthservres.biomedcentral.com/articles/10.1186/s12913-025-12381-w. 10.1186/s12913-025-12381-w PMC11812230 39930493

[24] Kirk MA, Kelley C, Yankey N, Birken SA, Abadie B, Damschroder L. A systematic review of the use of the Consolidated Framework for Implementation Research. Implement Sci. 2016;11:72. 10.1186/s13012-016-0437-z PMC4869309 27189233

[25] Palinkas LA, Horwitz SM, Green CA, Wisdom JP, Duan N, Hoagwood K. Purposeful Sampling for Qualitative Data Collection and Analysis in Mixed Method Implementation Research. Adm Policy Ment Health Ment Health Serv Res. 2015;42:533–44. 10.1007/s10488-013-0528-y PMC4012002 24193818

[26] Naderifar M, Goli H, Ghaljaie F. Snowball Sampling: A Purposeful Method of Sampling in Qualitative Research. Strides in Development of Medical Education. 2017;14.

[27] Malterud K, Siersma VD, Guassora AD. Sample Size in Qualitative Interview Studies: Guided by Information Power. Qual Health Res. 2016;26:1753–60. 26613970 10.1177/1049732315617444

[28] Ahmed SK. The pillars of trustworthiness in qualitative research. J Med Surg Public Health. 2024;2:100051.

[29] Naeem M, Ozuem W, Howell K, Ranfagni S. A step-by-step process of thematic analysis to develop a conceptual model in qualitative research. Int J Qual Methods. 2023;22. https://journals.sagepub.com/doi/10.1177/16094069231205789.

[30] Bingham AJ. From data management to actionable findings: a five-phase process of qualitative data analysis. Int J Qual Methods. 2023;22. https://journals.sagepub.com/doi/full/10.1177/16094069231183620.

[31] Redhead G, Lynch R. The unfairness of place: a cultural history of the UK’s ‘postcode lottery.’ Health Place. 2024;90:103301. 10.1016/j.healthplace.2024.103301 39293357

[32] Graley CEM, May KF, McCoy DC. Postcode lotteries in public health—the NHS health checks programme in North West London. BMC Public Health. 2011;11:738. 10.1186/1471-2458-11-738 PMC3195760 21955810

[33] Lyon RM, Cobbe SM, Bradley JM, Grubb NR. Surviving out of hospital cardiac arrest at home: a postcode lottery?. Emerg Med J. 2004;21:619–24. 15333549 10.1136/emj.2003.010363 PMC1726412

[34] Ajayi (Sotubo) O. A perspective on health inequalities in BAME communities and how to improve access to primary care. Future Healthc J. 2021;8:36–9. 10.7861/fhj.2020-0217 PMC8004339 33791458

[35] Huntley C, Loong L, Mallinson C, Bethell R, Rahman T, Alhaddad N, et al. The comprehensive English National Lynch Syndrome Registry: development and description of a new genomics data resource. 2024. Available from: www.thelancet.com. 10.1016/j.eclinm.2024.102465 PMC10864212 38356732

[36] Jasperson KW, Tuohy TM, Neklason DW, Burt RW. Hereditary and familial colon cancer. Gastroenterology. 2010;138:2044–58. 20420945 10.1053/j.gastro.2010.01.054 PMC3057468

[37] Biller LH, Ng K. The “scope” of colorectal cancer screening in Lynch syndrome: is there an optimal interval?. J Natl Cancer Inst. 2023;115:775–7. 37140568 10.1093/jnci/djad074 PMC10323891

[38] Castle-Clarke S, Hutchings R. Achieving a digital NHS: lessons for national policy from the acute sector. Available from: www.nuffieldtrust.org.uk/research.

[39] Asthana S, Jones R, Sheaff R. Why does the NHS struggle to adopt eHealth innovations? A review of macro, meso and micro factors. BMC Health Serv Res. 2019;19:984. 10.1186/s12913-019-4790-x PMC6925468 31864370

[40] Magrabi F, Lyell D, Coiera E. Automation in contemporary clinical information systems: a survey of AI in healthcare settings. Yearb Med Inform. 2023;32:115–26. 38147855 10.1055/s-0043-1768733 PMC10751141

[41] Webster EM, Perez L, Ahsan MD, Levi S, Chandler I, Thomas C, et al. Integration and usability of a digital cancer risk stratification tool to optimize identification of patients at risk for hereditary cancers: a pilot study. Gynecol Oncol. 2024;183:1–6. 38460222 10.1016/j.ygyno.2024.02.028

[42] Madanian S, Nakarada-Kordic I, Reay S, Chetty T. Patients’ perspectives on digital health tools. PEC Innov. 2023;2:100171. 10.1016/j.pecinn.2023.100171 PMC10294099 37384154

[43] Kerrison RS, Shukla H, Cunningham D, Oyebode O, Friedman E. Text-message reminders increase uptake of routine breast screening appointments: a randomised controlled trial in a hard-to-reach population. Br J Cancer. 2015;112:1005–10. 25668008 10.1038/bjc.2015.36 PMC4366892

[44] Yao R, Zhang W, Evans R, Cao G, Rui T, Shen L. Inequities in health care services caused by the adoption of digital health technologies: scoping review. J Med Internet Res. 2022;24:e34144 https://www.jmir.org/2022/3/e34144. 35311682 10.2196/34144 PMC8981004

[45] Usher-Smith JA, Hindmarch S, French DP, Tischkowitz M, Moorthie S, Walter FM, et al. Proactive breast cancer risk assessment in primary care: a review based on the principles of screening. Br J Cancer. 2023;128:1636–46. 36737659 10.1038/s41416-023-02145-w PMC9897164

[46] Van Ravesteyn NT, Miglioretti DL, Stout NK, Lee SJ, Schechter CB, Buist DSM, et al. What level of risk tips the balance of benefits and harms to favor screening mammography starting at age 40? $watermark-text $watermark-text $watermark-text. Ann Intern Med. 2012;156:609–17. http://cisnet.cancer.gov/. 22547470 10.1059/0003-4819-156-9-201205010-00002 PMC3520058

[47] Mills K, Paxton B, Walter FM, Griffin SJ, Sutton S, Usher-Smith JA. Incorporating a brief intervention for personalised cancer risk assessment to promote behaviour change into primary care: a multi-methods pilot study. BMC Public Health. 2021;21:205. 10.1186/s12889-021-10210-3 PMC7824918 33485309

[48] Kiran T, Rodrigues JJ, Aratangy T, Devotta K, Sava N, O'Campo P. Awareness and use of community services among primary care physicians. Healthc Policy. 2020;16:58. 32813640 10.12927/hcpol.2020.26290 PMC7435080

[49] Baynam G, Hartman AL, Catherine M, Letinturier V, Bolz-Johnson M, Carrion P, et al. Global health for rare diseases through primary care. Lancet Glob Health. 2024;12:e1192–9 10.1016/S2214-109X(24)00134-7 PMC13271179 38876765

[50] Stutzin Donoso F, Usher-Smith JA, Ficorella L, Antoniou AC, Emery J, Tischkowitz M, et al. Primary care online training on multifactorial breast cancer risk: pre-post evaluation study. BJGP Open. 2025. Available from: http://bjgpopen.org/lookup/doi/10.3399/BJGPO.2024.0305. 10.3399/BJGPO.2024.0305 PMC12728842 40234010

[51] Hindmarch S, Gorman L, Usher-Smith JA, Woof VG, Howell SJ, French DP. Development of a breast cancer risk assessment and primary prevention pathway for women aged 30–39 years: views of UK primary care providers on the role of primary care. PLoS ONE. 2024;19:e0308638. Available from: https://dx.plos.org/10.1371/journal.pone.0308638. 10.1371/journal.pone.0308638 PMC11398678 39269936

[52] Archer S, Donoso FS, Carver T, Yue A, Cunningham AP, Ficorella L, et al. Exploring the barriers to and facilitators of implementing CanRisk in primary care. Br J Gen Pract. 2023;73:E586–96. 37308304 10.3399/BJGP.2022.0643 PMC10285688

[53] Fusch P, Fusch GE, Ness LR. Denzin’s Paradigm Shift: revisiting triangulation in qualitative research. J Soc Change. 2018;10:2.

[54] Bellhouse S, Hawkes RE, Howell SJ, Gorman L, French DP. Breast cancer risk assessment and primary prevention advice in primary care: a systematic review of provider attitudes and routine behaviours. Cancers. 2021;13:4150. 10.3390/cancers13164150 PMC8394615 34439302

