# Supplementary material for: The breast cancer risk assessment pathway in England: a systems analysis of current challenges and ways to improve
Source: Br J Cancer. 2026 Jan 6;134(6):903–13. doi: 10.1038/s41416-025-03329-2 (PMC12960795; doi:10.1038/s41416-025-03329-2)

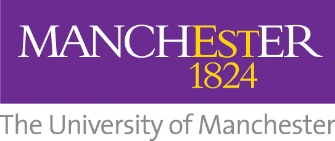

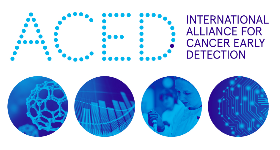

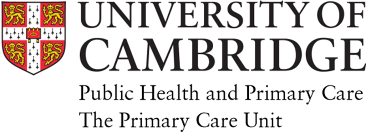


**PREVENT-Breast** Systems-based analysis of breast cancer risk assessment and screening

**Interview guide**

1. **Opening questions**
   1. Could you please describe your professional role?
   2. How is your role associated with breast cancer risk assessment or screening?
2. **The current system: Risk assessment pathway and Screening programme**

I/we would like to start by showing you an outline of what we think the current system of breast cancer risk assessment and subsequent management is for women under the age of 50. We have split this into three parts: how women access breast cancer risk assessment, the risk assessment process itself, including the risk factors used, and then the risk categorisation and subsequent management process.

*For each of the three parts, show the map of the current pathway (slides 1, 2 and 3: pages 4-6 of the current) and ask the following questions:*

- 1. Does this match with your understanding of the process in your region? [Prompt for risk assessment – do you think risk assessment is happening at all these opportunities?]
  2. Have any key aspects been missed?
  3. Do you think there are any challenges and/or limitations of this current system/pathway?
  4. How would you describe the current system/pathway in terms of access [and equity]?
- Are there any groups of women that you think might not be included in the risk assessment process, and why do you think that is?

1. **Design of a new proactive breast cancer risk assessment pathway**

Having discussed and described the current system, I/we would now like to focus on ways in which we could improve the pathway.

- 1. Which areas do you think need improvement most and why?

*Starting with the area that the interviewee thinks is most important, show the current map again and talk through potential options for improvement (pages 4-7 of the current).*

1. ***Access to breast cancer risk assessment*** (barriers and facilitators)
2. What do you think is the best and most feasible way to improve [equity of] access for women to risk assessment of breast cancer?
3. We have identified a few ways that women could be provided access to or proactively invited for risk assessment for breast cancer (*read or show them the options*). Which do you think is the most promising and why? Are there any you would definitely not choose and why?

*Examples of options for alternative approaches for access – show slide 4*

- - *Offer to women alongside NHS Health Checks at age 40 or annual health checks for those with learning disabilities or chronic mental health conditions*
  - *Offer to women alongside cervical screening at screening appointment closest to a given age (e.g. 35)*
  - *Invite all women at a given age (e.g. 35 or 40) for a risk assessment via letter from GP practice*
  - *Offer to all women over a given age (e.g. 35 or 40) through religious groups, community centres or other third-sector organisations*
  - *Offer to all women over a given age (e.g. 35 or 40) through pharmacies*
  - *Offer to women presenting to secondary care with breast symptoms*
  - *Ask all women diagnosed with breast cancer to encourage their female relatives to contact their GP for risk assessment*
  1. ***The risk assessment process and factors used for initial assessment in primary care*** *(barriers and facilitators)*

As we discussed earlier, at the moment the role of primary care is to triage women based on their first- and second-degree family history and to refer women who meet referral criteria into family history or genetics clinics. In those clinics a full risk assessment is then done which then includes a more comprehensive family history and potentially PRS and genetic testing and reproductive history etc.

1. Do you think primary care should continue to have a triage role or should primary care take on a larger role in the risk assessment? Why?

Alternatives include (a) primary care doing the full risk assessment, including PRS and reproductive history etc, for all women, or (b) for primary care to continue to have a triage role but include risk factors beyond family history that are currently used in family history or genetics clinics into that triage assessment (*illustrate on map – slides 5&6, pages 8-9 of the current*)

1. Could you please describe any advantages or disadvantages of any of those approaches? [short vs long term]
2. Who do you think is best placed in the system to lead on the full risk assessment?
   1. **The risk categorisation process and subsequent management** (what should happen next? Who should be referred for further screening and where?)
      1. If access to breast cancer risk assessment is improved, would there be capacity within your area of the pathway for an increase in women at moderate or high risk of breast cancer?
      2. How would you see the role of your area of the pathway changing or needing to change?
      3. If primary care were to take a larger role in risk assessment, one option might be for women to be referred directly from primary care into breast screening services (show slide 6)
         1. Do you think all women at above-population level risk need to be seen/reviewed in family history/clinical genetics clinics before being referred for enhanced screening? Why? Why not?
         2. What about for tamoxifen/anastrozole? Do you think primary care should be able to prescribe tamoxifen/anastrozole for primary prevention without review through family history/clinical genetics clinics?
      4. How often do you think risk should be reassessed / what should trigger that reassessment and who should lead on that risk assessment?
      5. Do you think breast cancer should have its own distinct program for (screening and) risk assessment, or is there a better approach?

So far, we have considered a full risk assessment to be a comprehensive family history, other risk factors such as age, reproductive history etc. There is also increasing evidence that mammographic density is important in risk assessment.

1. Where do you think mammographic density fits into the pathway?
2. Do you think all women should be offered a standalone mammogram e.g. at age 40 for breast density assessment? Why/why not?
3. Do you think risk should be reassessed each time a woman has a mammogram, either within screening or through breast services after presenting with a breast concern? Why/why not?
4. **General questions**
   1. Is there anything else we need to take into consideration regarding the above in order to develop a new risk assessment pathway?
   2. Short-term vs. long-term improvement: What could be done in the near future, and what could we aim for in the long term?
   3. How ready do you think the system is to implement a new pathway?
   4. Who would be key stakeholders to involve in this interview study and the co-design study? Do you have any recommendations of people we should contact?


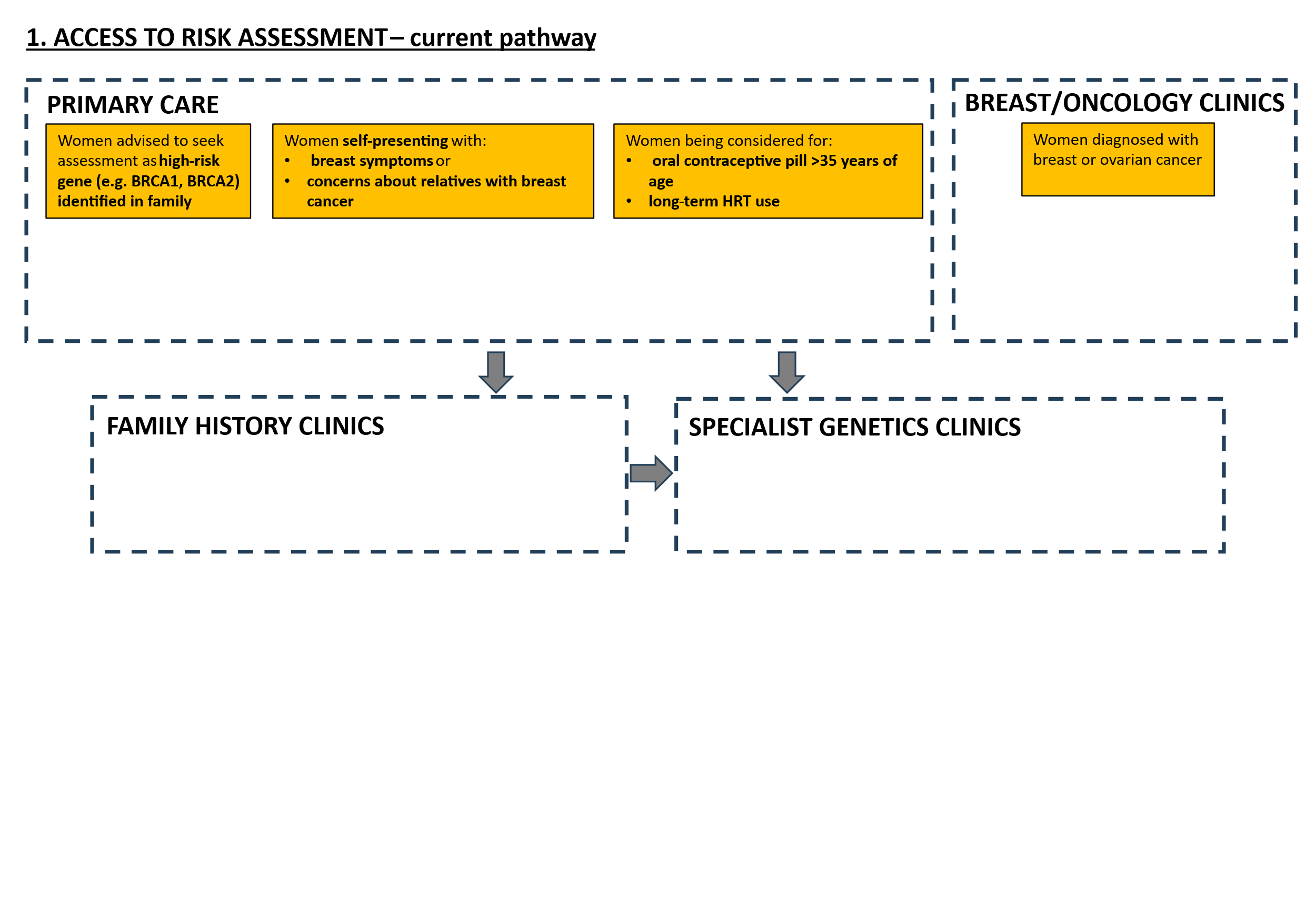


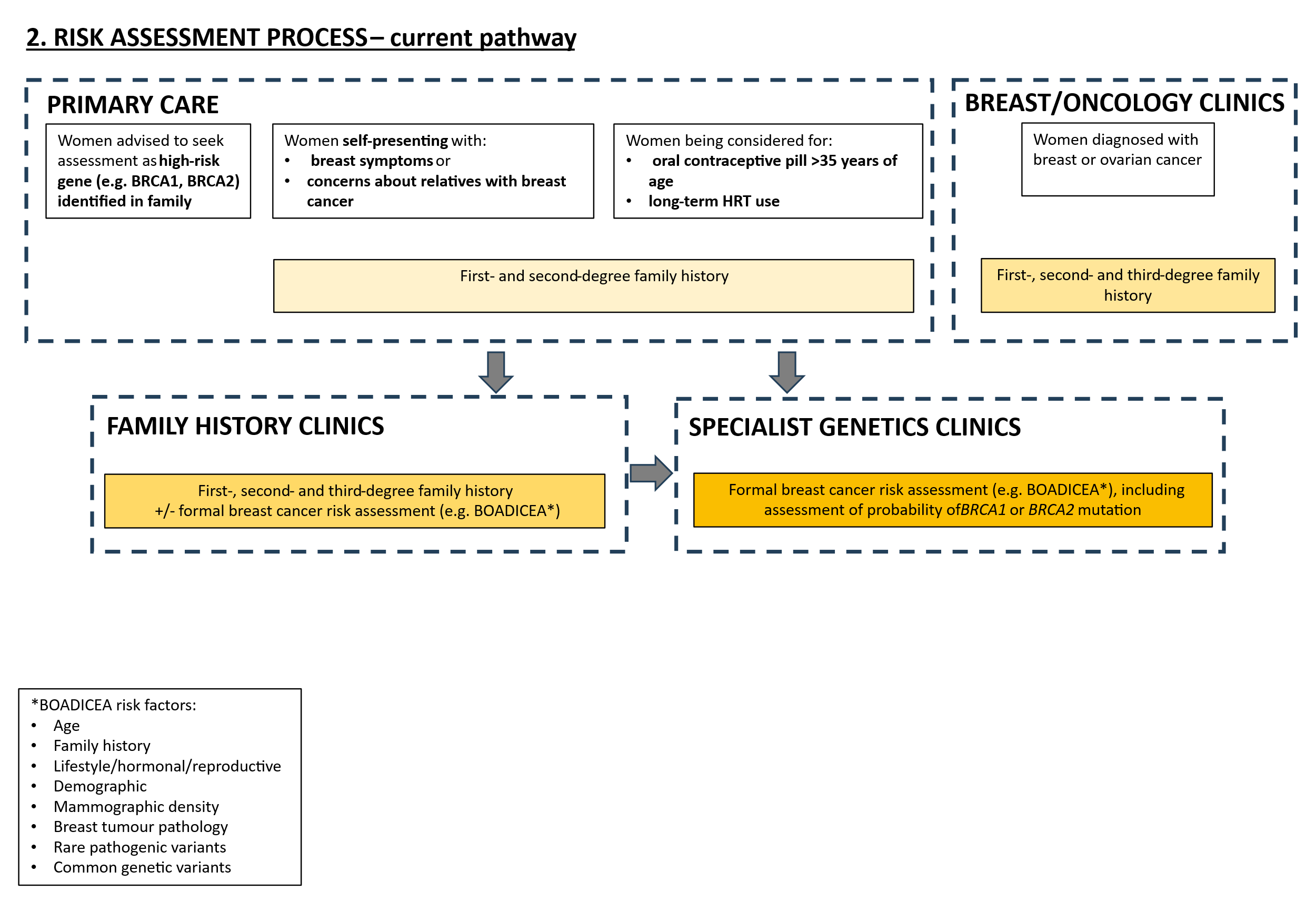


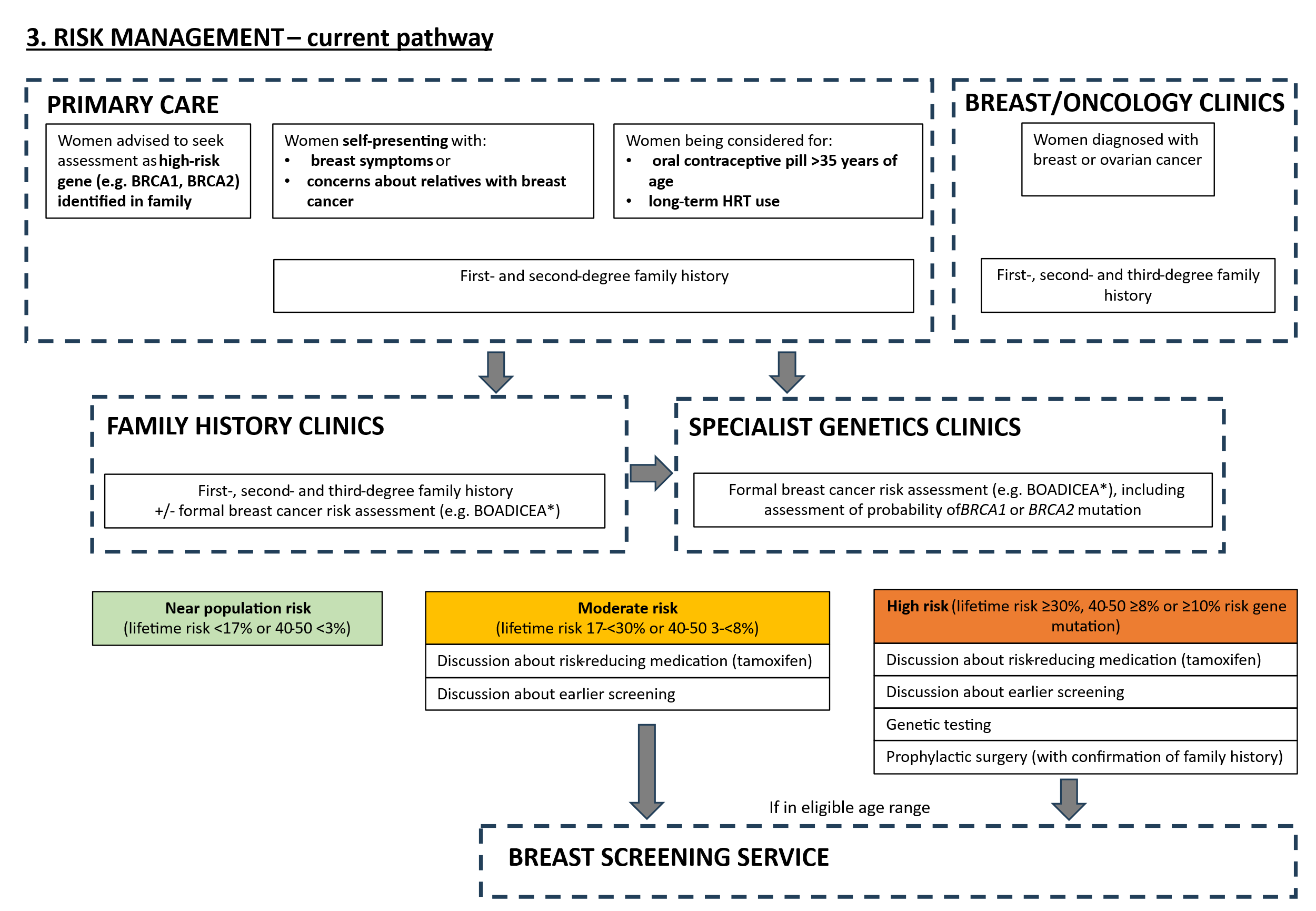


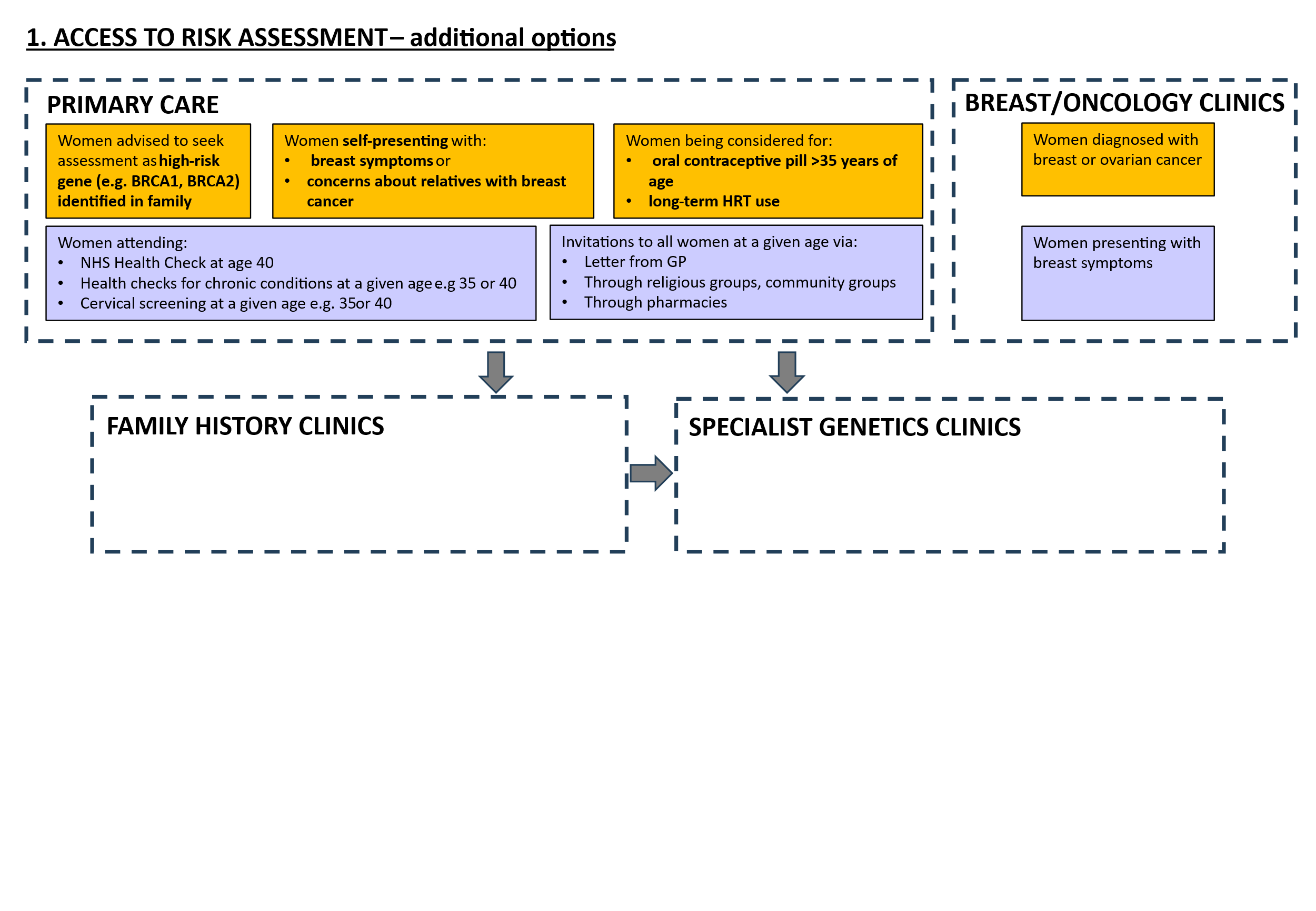


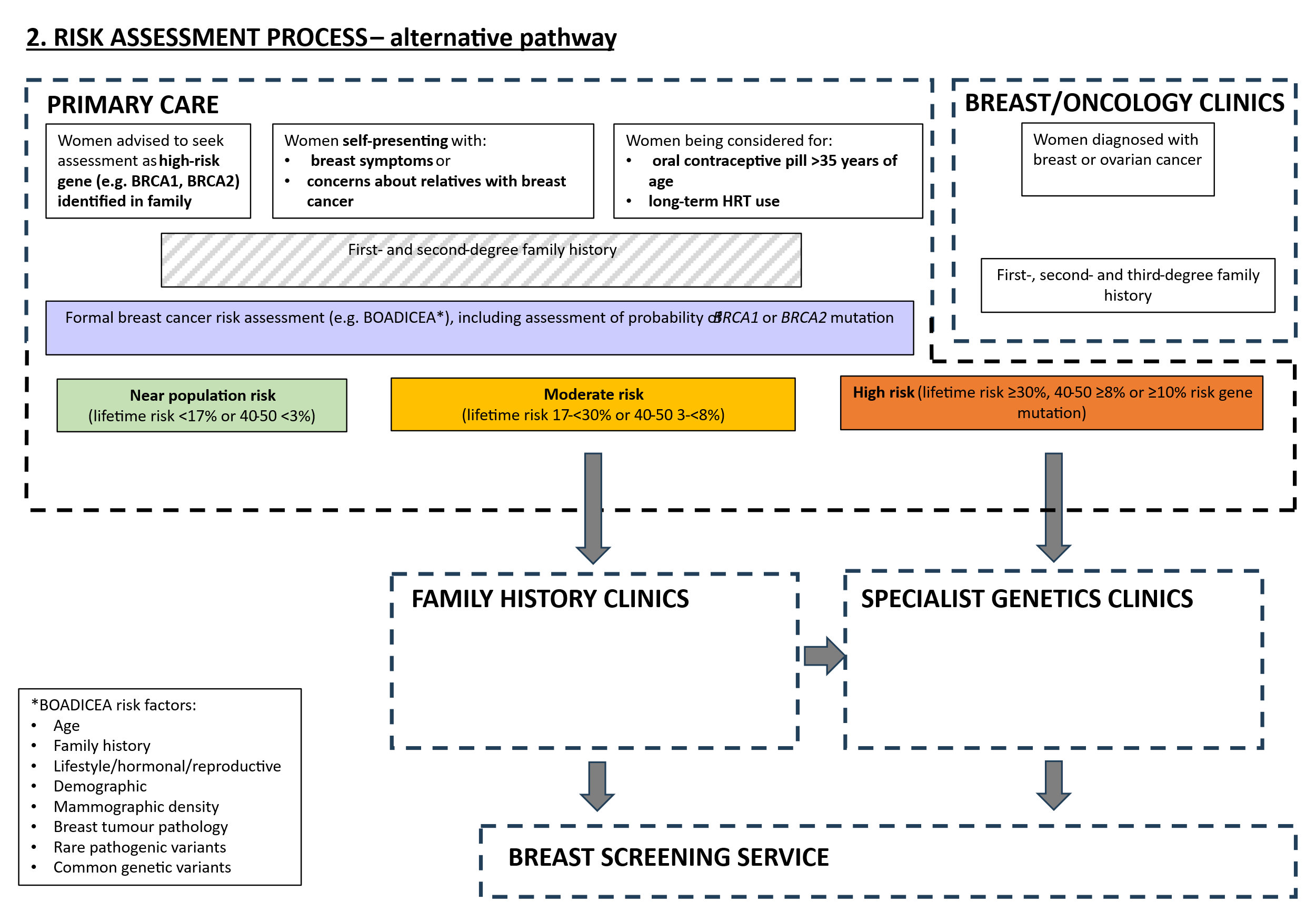


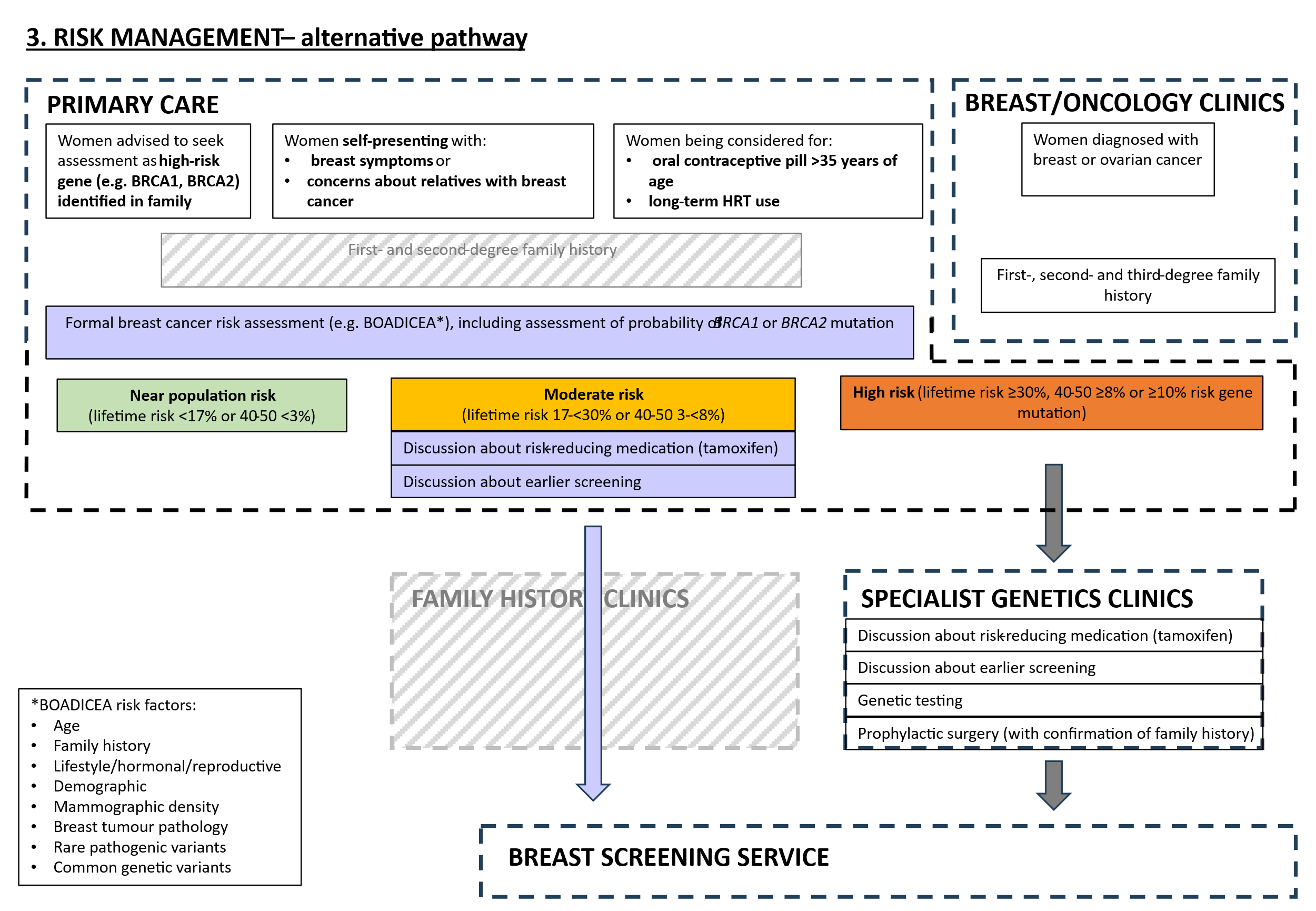

Supplement: Supplementary file 1 — Interview Guide [file 41416_2025_3329_MOESM1_ESM.docx]
